# T-cell mediated immune response to HIFU-induced liquefaction of murine B16 melanoma

**DOI:** 10.1186/2050-5736-3-S1-O43

**Published:** 2015-06-30

**Authors:** Tatiana Khokhlova

**Affiliations:** 1 University of Washington, Seattle, Washington, United States

## Background/introduction

Anti-tumor immune response caused by High Intensity Focused Ultrasound (HIFU) therapy has been a subject of controversy. Most agree that the response is more likely to be induced by mechanical, rather than thermal effects of HIFU. The goal of the current work was to study the effect of HIFU-induced liquefaction of a tumor on tumor-specific and non-specific T-cell mediated immune response in a mouse model.

## Methods

B16 melanoma was inoculated in a hind limb of B6 wild type mice. When the tumor reached the diameter of 1 cm, it was treated with HIFU optimized for boiling histotripsy (BH) - a technique utilizing millisecond-long pulses to create boiling bubbles via rapid shockwave heating. The interaction of shocks with the ensuing vapor cavity fractionates tissue into subcellular debris with negligible thermal effect. The control group received sham treatment. Groups of animals were sacrificed 2 and 7 days post treatment, and the lymphatic organs, blood and tumor tissue were analyzed by flow cytometry for T cell and dendritic cell activation status, phenotype, specificity and reactivity.

## Results and conclusions

Although BH delayed the tumor growth compared to the control group, it did not change subsequent growth rate. No statistically significant difference from the control group was found in neither the number, nor the phenotype and activation of cytotoxic, helper and regulatory T cells (neither tumor-specific, nor non-specific). Activation status of the dendritic cells was also unaltered by the treatment. However, a two-fold difference in the number of non-dendritic cells bearing MHCII receptor was found in the spleen and inguinal lymph nodes suggesting a humoral response. These results indicate that T cell mediated mechanism is unlikely to be triggered by BH alone, but warrant further investigation of the systemic effects of this unique treatment modality.

